# Supplementary figures and images for: Sex- and tissue-specific expression of odorant-binding proteins and chemosensory proteins in adults of the scarab beetle Hylamorpha elegans (Burmeister) (Coleoptera: Scarabaeidae)
Source: PeerJ. 2019 Jun 12;7:e7054. doi: 10.7717/peerj.7054 (PMC6571001; doi:10.7717/peerj.7054)

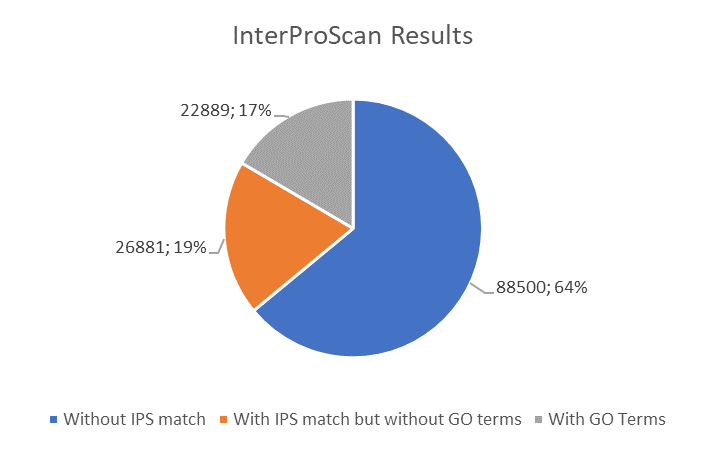

Supplement: Figure S1 [file peerj-07-7054-s001.png]
