# Supplementary material for: Sex- and tissue-specific expression of odorant-binding proteins and chemosensory proteins in adults of the scarab beetle Hylamorpha elegans (Burmeister) (Coleoptera: Scarabaeidae)
Source: PeerJ. 2019 Jun 12;7:e7054. doi: 10.7717/peerj.7054 (PMC6571001; doi:10.7717/peerj.7054)
Supplement: Figure S2 — Amino-acidic sequence alignment was performed using ClustalW. Red asterisks indicate conserved Cysteine residues. [file peerj-07-7054-s002.pdf]

Figure S2. Alignment of OBP sequences

```
                    10         20         30         40         50         60         70
            ....|....| ....|....| ....|....| ....|....| ....|....| ....|....| ....|....|
HeleOBP2    ---------- ---------- ---------- ---------- ---------- ---------- ----------
HeleOBP1    ---------- ---------- ---------- ---------- ---------- ---------- ----------
HeleOBP5    ---------- ---------- ---------- ---------- ---------- ---------- ----------
HeleOBP7    ---------- ---------- ---------- ---------- ---------- ---------- ----------
HeleOBP9    ---------- ---------- ---------- ---------- ---------- ---------- ----------
HeleOBP10   ---------- ---------- ---------- ---------- ---------- ---------- ----------
HeleOBP11   ---------- ---------- ---------- ---------- ---------- ---------- ----------
HeleOBP3    ---------- ---------- ---------- ---------- ------MCLS LTIFVRPAPY KQLPISNKPH
HeleOBP6    ---------- ---------- ---------- ---------- ---------- ---------- ----------
HeleOBP13   ---------- ---------- ---------- ---------- ---------- ---------- ----------
HeleOBP14   ---------- ---------- ---------- ---------- ---------- ---------Y DFGDEFFNQL
HeleOBP15   ---------- ---------- ---------- ---------- ---------- ---------- -LECELSSNQ
HeleOBP16   ---------- ---------- ---------- ---------- ---------- ---------- ----------
HeleOBP4    ---------- ---------- ---------- ---------- ---------- ---------- ----------
HeleOBP18   ---------- ---------- ---------- ---------- ---------- ---------- ----------
HeleOBP19   ---------- ---------- ---------- ---------- ---------- ---------- ----------
HeleOBP21   ELTPTANLTL ETAHYTCLVG SGIELNQIND SLNGNYSENK QVAQYFQCMF RMYGLLDVHG NLKNETIFDA
HeleOBP23   ---------- ---------- ---------- ---------- ---------- ---------- ----------

                     80         90        100        110        120        130
140                                                                              *
            ....|....| ....|....| ....|....| ....|....| ....|....| ....|....| ....|....|
HeleOBP2    ---------- -------DFL DKANERMAKV ---------- ---------- ---------- --FDECTTSA
HeleOBP1    ---------- ------QDSA EDRQERIRKY ---------- ---------- ---------- --REECVEET
HeleOBP5    ---------- -----VPKLQ TLFQEHAFKQ ---------- ---------- ---------- --GSECLSEV
HeleOBP7    ---------- -----RPDDL VNNEKTCLES ---------- ---------- ---------- --STTFFEKI
HeleOBP9    ---------- -----VTRIQ EGIKGGIATS ---------- ---------- ---------- --GAICLTEI
HeleOBP10   ---------- -----VTRIQ EGIKGGIATS ---------- ---------- ---------- --GAICLTEI
HeleOBP11   ---------- ---ETIQEHG KRVLEKIMDT ---------- ---------- ---------- --GASCAEKL
HeleOBP3    YNADSRTTLK VFISFLQENP TRRRMKHTKM NFFALLFLSV A-----LIRE ITSAETSSDA LRRKICIQQT
HeleOBP6    ---------- ---QTYDEEK QKLRQQAMNT ---------- ---------- ---------- --LGECKTKV
HeleOBP13   ---------- ---------- --YLEQAIAA ---------- ---------- ---------- --ADGCLGDL
HeleOBP14   LTREYDDFSS VDSAFLHPRA RRDEEAAKCH HKHK------ ---------- ---------F CCADELMFQL
HeleOBP15   NQEDIKRFTN MCMTKTVTKT DEFTENASAE QSYEN----- ---------- ---------- SYEDEAHSSL
HeleOBP16   ---------- ------EDAD TDLLIGSDAQ ---------- ---------- ---------- --RNQCIKEL
HeleOBP4    ---------- --------KP PPFMDDMHQY ---------- ---------- ---------- --RDDCLKDL
HeleOBP18   ---------- ---------- --DMTAVSAA ---------- ---------- ---------- --MHDCLTKL
HeleOBP19   ---------- ---------- ---FAGIDQY ---------- ---------- ---------- --KDLCSREL
HeleOBP21   IHSLEKEHSY TEDGVLNCQN DTFNRNITRN EIAYEFFKCF KNYTETAYYW SILKYLLNKQ GAYPAITLTQ
HeleOBP23   ---------- ---------- ------NDAG ---------- ---------- ---------- --RIACINKL

                    150        160        170        180        190        200
210                                                    *    *
            ....|....| ....|....| ....|....| ....|....| ....|....| ....|....| ....|....|
HeleOBP2    GATKDDIM-- -----ELMEI RIPSRKEAK- ---------C VLACYHKKYG IQDQDGKLDK TAAIEAMKDL
HeleOBP1    KVDPTLID-- -----KADAG DFSDTKELK- ---------C FAKCFYMKAG FINEQGELLM DVVK------
HeleOBP5    GATMDELK-- -----SIVKQ DIPTTRAGM- ---------C LITCIHEKFG MQDNNGKMLR DGTLAFLEQV
HeleOBP7    QHQATGLFTS AKRKFGIDPN AQDEPLNCD- ----------  YYLCILRSIG MVNDYGILEL EGTKLWLN--
HeleOBP9    EATMDDLR-- -----NLVEH VKPTTRAEM- ---------C LITCVHKKVG MQNEQGKLVE AGISNFFAPL
HeleOBP10   EATMDDLR-- -----NLVEH VKPTTRAEM- ---------C LITCVHKKVG MQNEQGKLVE AGISNFFAPL
HeleOBP11   GATPEDME-- -----KLMKK ELPDSKAAQ- ---------C VISCVNKAFQ LQNEDGSINK GVMPPSMADI
HeleOBP3    NVNPSLVD-- -----KANEG QFSDKRELQ- ---------C YFRCYYLESG FINDSGEIQT DIIK------
HeleOBP6    GASDEDIQ-- -----AIVNK QLPTTKAGL- ---------C MLECGFSTVG IMTN-GKLDS ANTLKILAPA
HeleOBP13   GLDASELK-- --DLVQKMDN LLEPTHDGK- ---------C LVACSMQAAG FMKDYKYNED A---------
HeleOBP14   HDKYRDIKRE CYKEVTGKEF GGGPPFTCEE LEERKKEMRC VAECAGKKKG MIDDKGNLKE EEAKELVKAI
```

```
HeleOBP15  EEIPDARALS KGNISNSTKS TINNTRDGEN LNNTEITNDC VVQCVLKQLG MVDPSGYPDH VKISEN-LMK
HeleOBP16  KFNADEIR-- ---EIDSLDD LDDMTMNEK- ---------C ILRCVLMKLG AIDKQGALVP D---------
HeleOBP4   GLPADTATHP PPSADGGPPS NNEPSHDMK- ---------C LLNCLLRKGG ILNSSGVLQP D---------
HeleOBP18  NIDLKEVN-- --AMENAVMT NTEPSRDGK- ---------C LASCTMEKAG LTKDGKIIVD A---------
HeleOBP19  NVSVSNWQRE PTSPEGKPTY LAEPDHGGK- ---------C LLNCMMLKAG VIDETGAIQ- ----------
HeleOBP21  SLKLETVHFT CAMGSDVQLQ SIKNSLNAIY TDDNK--LTL YFQCVFRMYG YVDKDGSLKN ETMLN-----
HeleOBP23  NLNPDEIR-- ---GVDALET LEVLTKDEK- ---------C VLRCMLMELG TIDSQGAIAP A---------

                   220        230        240        250        260        270
280
                            *               *          *
           ....|....| ....|....| ....|....| ....|....| ....|....| ....|....| ....|....|
HeleOBP2   KVEDPELYDK AVQLFDTCIE QVPNQ----K CECETA-AIF MYCFNIYGKM MGLKPGMVPM ----------
HeleOBP1   EKIPPEHDRE KALAIIELCK NLKE-----S DTCETA-YAI HKCYFQNAHA ANLHKN---- ----------
HeleOBP5   KD-DPAYYEL VKEHFLYCLD TVSNN----D EKCTIG-NNF MSCLVLGGRK KGIFD----- ----------
HeleOBP7   RNVPQEYNNE MVEHATLCFQ NIGKNNVN-E TNCDRS-PKY IKCLHNYEKC QVFKFP---- ----------
HeleOBP9   MEADSDYFAV SKQHFLDCAK TVPDD----D DECVIG-ARF NDCVIIGGKE KGLLD----- ----------
HeleOBP10  MEADSDYFAV SKQHFLDCAK TVPDD----D DECVIG-ARF NDCVIIGGKE KGLLD----- ----------
HeleOBP11  KGIDEDIYNK MATVWDICSQ KAVGS----G DECDAG-IHL VKCMKEESEK LGLTKEAMGF ----------
HeleOBP3   SKIPQKLDRK TAQQAIDTCK KVKG-----L DSCETA-YEL QKCLYDN--K VKL------- ----------
HeleOBP6   LSKNEDKSKK VTEALNNCEK EVGNGG---A DGCETA-KLI AECFKKESMK SA-------- ----------
HeleOBP13  -IMKSLPGTA S-VKFDNCQD -IMD-----T DDCDRY-FKM SVCVTKQISI NE-------- ----------
HeleOBP14  TADLGWFQSV SDEIITKCAG EAKAAAEKHK DGCNPSDLKF AFCIFKEIQL NCPADQIKDQ ARCEAMRESI
HeleOBP15  GIDNRELKDF LQDSTDDCFQ LMEQDEH--V DHCYFS-TQL IKCLAEKGKS NCGDWPMTDL PFHHIL----
HeleOBP16  -ELDEQITKD LNIDFSKCVP -QKNI----T DPCEQT-YVL TRCMFQLLFK AAEKSA---- ----------
HeleOBP4   -KVKLSVDAS V-VDLNKCTT -VTD-----S DPCQQT-YLI ERCIMDQLPK QSM------- ----------
HeleOBP18  -VNALDPTIA S-VDLQQCQG -LTG-----S DNCERD-YNI LKCGVGQYML KYKSKPQQ-- ----------
HeleOBP19  -----SLFIY L-PDSHKCAN -ITD-----T DVCQRG-YLI ESCIRKELRL AVRL------ ----------
HeleOBP21  SIPSEDKDKS YTEDAIKSCQ YVWTP----- TSDAIA-YEF FKCFRNKTDA AAYWTTLKEL SSKNDKQDVS
HeleOBP23  -VLDRKMLKD LKIDFSICVP -QKNI----T DLCEQT-HVL TQCLMKLSVG AVQKS----- ----------

           ....|....
HeleOBP2   ---------
HeleOBP1   ---------
HeleOBP5   ---------
HeleOBP7   ---------
HeleOBP9   ---------
HeleOBP10  ---------
HeleOBP11  ---------
HeleOBP3   ---------
HeleOBP6   ---------
HeleOBP13  ---------
HeleOBP14  KKYDHPPVH
HeleOBP15  ---------
HeleOBP16  ---------
HeleOBP4   ---------
HeleOBP18  ---------
HeleOBP19  ---------
HeleOBP21  NVLVDFLL-
HeleOBP23  ---------
```
